# Maternal–Fetal Transfer of Anti-SARS-CoV-2 Antibodies in Amniotic Fluid: Insights from Maternal Vaccination and COVID-19 Infection

**DOI:** 10.3390/jcm13175023

**Published:** 2024-08-25

**Authors:** Inshirah Sgayer, Marwan Odeh, Meital Gal-Tanamy, Mona Shehadeh, Hagai Rechnitzer, Yousef Haddad, Rudi Hamoudi, Nisreen Kinaani Mousa, Vivian Abu Uksa Dakwar, Maya Frank Wolf, Tzipora C. Falik Zaccai, Lior Lowenstein

**Affiliations:** 1 Department of Obstetrics and Gynecology, Galilee Medical Center, Nahariya 22000, Israel; marwano@gmc.gov.il (M.O.); usef.88@gmail.com (Y.H.); hamoudirudi91@gmail.com (R.H.);; 2 Azrieli Faculty of Medicine, Bar Ilan University, Safed 13100, Israel; mgtanamy@gmail.com (M.G.-T.); monas@gmc.gov.il (M.S.); hagair@gmc.gov.il (H.R.);; 3 Clinical Laboratories Division, Galilee Medical Center, Nahariya 22000, Israel; 4 Clinical Microbiology, Galilee Medical Center, Nahariya 22000, Israel; 5 Institute of Human Genetics, Galilee Medical Center, Nahariya 22000, Israel; nisreenk@gmc.gov.il (N.K.M.); viviana@gmc.gov.il (V.A.U.D.)

**Keywords:** COVID-19, SARS-CoV-2, amniocentesis, maternal immunity, antibody transfer, booster

## Abstract

**Objectives**: As the COVID-19 pandemic wanes, understanding maternal–fetal antibody transfer remains crucial for optimizing vaccination strategies. This study evaluates anti-SARS-CoV-2 antibody levels in amniotic fluid following maternal BNT162b2 mRNA vaccination and/or COVID-19 infection during early pregnancy, focusing on the first and second trimesters. **Methods**: A retrospective cohort study was conducted at a tertiary university-affiliated hospital, involving 149 pregnant women who underwent amniocentesis. Anti-SARS-CoV-2 spike IgG levels were measured in amniotic fluid samples. Participants were categorized based on vaccination and infection status: vaccine-only, infection-only, vaccine + infection, and no vaccine/infection. Correlations between antibody levels and the time since vaccination or infection were analyzed. **Results**: The vaccine + infection group had a higher proportion of positive antibody levels compared to the vaccine-only group (63.6% vs. 35.9%, *p* = 0.029). Median SARS-CoV-2 IgG levels were significantly higher in the vaccine + infection group (283.0 AU/mL) than in the vaccine-only group (64.1 AU/mL, *p* = 0.006). Women who received three vaccine doses had higher antibody levels and more positive antibody rates compared to those with one or two doses. A significant negative correlation was found between antibody levels and the interval since the last vaccine dose or infection. **Conclusions**: Our results indicate the presence of anti-SARS-CoV-2 antibodies in the amniotic fluid, reflecting antibody transfer during early pregnancy. However, a noticeable decrease in immunity was observed, as indicated by declining amniotic fluid antibody levels over time. Further studies are needed to determine the optimal timing and number of boosters required to protect against new variants of SARS-CoV-2.

## 1. Introduction

While the World Health Organization has declared that the novel coronavirus disease (COVID-19), caused by severe acute respiratory syndrome coronavirus 2 (SARS-CoV-2), is no longer a global health emergency, the pandemic is not over. Pregnant women are at greater risk for severe COVID-19 infection than nonpregnant reproductive-aged females [1,2]. Unvaccinated compared to vaccinated pregnant women are susceptible to a worse clinical course, including a higher risk of critical care admission and perinatal death [3,4,5]. The COVID-19 vaccine is deemed to be effective and safe during pregnancy, reduces the risk of maternal SARS-CoV-2 infection, and provides neonatal protection [6,7].

Data regarding maternal-fetal transfer of antibodies against SARS-CoV-2 after periconceptional or first-trimester vaccination are based on umbilical cord blood antibody levels determined after delivery [8]. Rottenstreich et al. found that maternal antibodies after delivery were the lowest after first-trimester vaccination and the highest after third-trimester vaccination. The neonatal antibody levels showed a similar pattern [7].

Although the optimal timing for maternal vaccination has yet to be determined, COVID-19 vaccination to reduce the risk of SARS-CoV-2 infection is recommended for unvaccinated women planning pregnancy or early in their pregnancy [9]. Data are limited regarding maternal-fetal SARS-CoV-2 antibody transfer in early pregnancy. Determining antibody levels in amniotic fluid could aid in understanding the kinetics of the maternal response to COVID-19 infection or vaccination [10]. Toward this goal, we determined SARS-CoV-2 antibody levels following maternal BNT162b2 mRNA vaccination or COVID-19 infection in amniotic fluid samples taken during amniocentesis.

## 2. Methods

This retrospective cohort study included pregnant women who underwent a genetic amniocentesis during the year 2021 at the Galilee Medical Center, a tertiary-care university affiliated hospital in northern Israel. This study aimed to evaluate the presence of anti-SARS-CoV-2 IgG antibodies in the amniotic fluid of pregnant women who underwent genetic amniocentesis. Amniotic fluid was obtained from residual specimens of women who had banked amniotic fluid samples in the cytogenetic laboratory at the Institute of Human Genetics at our institution.

Amniocentesis was performed according to our standard protocol under ultrasound guidance, and the specimens were transported immediately to the cytogenetic laboratory at room temperature. The amniotic fluid specimens were centrifuged at 1200 rpm for 8 min and used for genetic testing. Any residual specimens were stored at −20 °C temperature and used for this study.

A total of 301 women underwent amniocentesis during the study period. After a review of the electronic medical records, we excluded 130 women who did not have any residual specimens stored in the cytogenetic laboratory. Accordingly, 166 women were included in the study. The primary exposures of interest were maternal SARS-CoV-2 vaccination status and/or infection. This study specifically focused on the timing of the last vaccine dose or infection relative to the amniocentesis procedure. Women were categorized into four groups: (1) vaccinated only, (2) infected only, (3) both vaccinated and infected, and (4) neither vaccinated nor infected.

Demographic and clinical data were collected from our electronic databases, including maternal age, gravidity, parity, the use of assisted reproductive technology, the presence of medical conditions, medication use, body mass index (BMI), and gestational age at the time of amniocentesis. Specific data on COVID-19 exposure, including the number of vaccine doses received (one, two, or three doses) and whether the women was accessed, and the SARS-CoV-2 IgG levels in amniotic fluid were categorized as negative, borderline, or positive according to these intervals and the women’s exposure history. During the study period in 2021, the primary COVID-19 vaccines available in Israel were the Pfizer-BioNTech (BNT162b2) and Moderna (mRNA-1273) vaccines. The majority of the vaccinated women included in this study likely received the Pfizer-BioNTech vaccine, which was the most widely administered vaccine at the time.

### 2.1. Laboratory Methods

Anti-SARS-CoV-2 spike IgG levels were measured in amniotic fluid samples using the SARS-CoV-2 IgG II Quant assay, a 2-step chemiluminescent microparticle immunoassay for the quantitative determination of IgG antibodies to SARS-CoV-2 on Abbott Architect i1000SR immunoassay analyzer (Abbott Park, IL, USA). The SARS-CoV-2 IgG II Quant assay is designed to detect IgG antibodies, including neutralizing antibodies, to the receptor-binding domain of the S1 subunit of the spike protein of the SARS-CoV-2 virus in human serum and plasma. According to the kit datasheet, antibody levels of >150 AU/mL are considered positive, while levels in the range of 50–150 AU/mL are considered borderline [11].

### 2.2. Ethics

This study was conducted according to the guidelines of the Declaration of Helsinki and was approved by the Institutional Ethics Committee of the Galilee Medical Center (protocol NHR-021-111, date of approval 11 January 2022).

### 2.3. Statistics

Continuous variables are presented as medians and ranges. Qualitative variables are presented as frequencies and percentages.

Data of vaccinated, infected, infected + vaccinated, and not vaccinated or infected women were compared with the Kruskal–Wallis and Mann–Whitney tests. The correlation between antibody levels and the interval from the last vaccine dose/infection was analyzed with Spearman’s correlation coefficient test due to the non-normal distribution shape. A logarithmic transformation for antibody levels was used due to the non-normal distribution.

Kaplan–Meier survival curves were presented for the relation of positive antibody levels (>150 AU/mL) to the interval from the last vaccine dose or infection to amniocentesis. The groups were compared using the log-rank test. A *p*-value < 5% was considered significant. The analyses were performed using IBM statistics, version 26 (SPSS).

## 3. Results

This study included 166 women who underwent amniocentesis during the study period. Five women had twin pregnancies and twelve underwent amniocentesis during the third trimester. Due to the limited data on SARS-CoV-2 antibodies in the amniotic fluid, these 17 women were not excluded, and their results are reported separately.

### 3.1. Women with Singleton Pregnancies Who Underwent Amniocentesis during the Second Trimester

In total, 149 women underwent amniocentesis during the second trimester. Their background data are presented in Table 1. No significant correlations were observed of SARS-CoV-2 IgG antibody levels with maternal age, parity, gestational age at amniocentesis, or BMI. In total, 92 women were vaccinated against SARS-CoV-2 before amniocentesis (vaccine-only group), 8 had been infected with COVID-19 before amniocentesis (infection-only group), 22 had been infected with COVID-19 and also vaccinated against SARS-CoV-2 (vaccine + infection group), and 27 had not been vaccinated or infected (no vaccine/infection group). The characteristics of the four groups are described in Table 2. The duration from the last vaccine dose/infection to amniocentesis was similar for the vaccine-only and the vaccine + infection groups (11.0 and 10.7 weeks, respectively, *p* = 0.699). These values were significantly lower (*p* < 0.001) than for the infection-only group, 42.3 weeks.

The proportions of women vaccinated/infected in the 6 months before amniocentesis were similar between the vaccine-only and vaccine + infection groups (78.3% and 81.8%, respectively), and higher (*p* < 0.001) than that of the infected-only group, 12.5%. Among the women who were vaccinated/infected in the 6 months before amniocentesis, the duration from the last vaccine dose/infection to amniocentesis was similar between the vaccine-only and vaccine + infection groups (8.9 and 9.8 weeks, respectively, *p* = 0.09).

**Figure 1:**
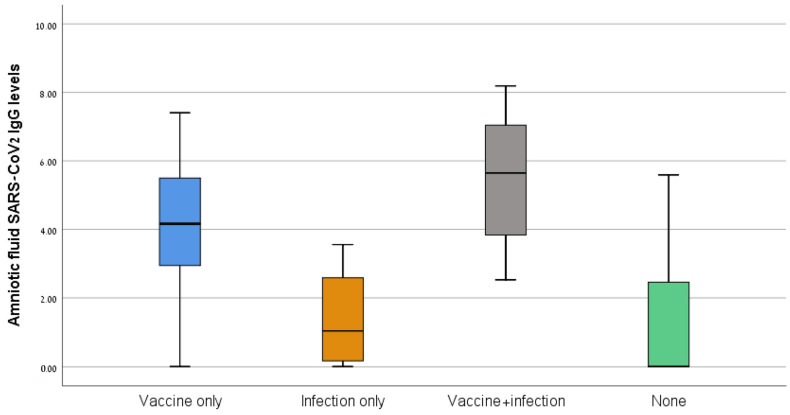
SARS-CoV-2 IgG levels of the four study groups.

The median antibody levels differed significantly between the four groups (64.1 AU/mL for the vaccine-only group, 2.9 AU/mL for the infection-only group, 283.0 AU/mL for the vaccine + infection group, and 17.9 AU/mL for the no vaccine/infection group, *p* < 0.001) (Figure 1). The median antibody levels were higher in the vaccine + infection than the vaccine-only group (283.0 vs. 64.1 AU/mL, *p* = 0.006). For the sub-group of women who had been vaccinated/infected in the 6 months before amniocentesis, the median antibody levels were higher in the vaccine/infection than the vaccine-only group (317.5 vs. 217.5 AU/mL, *p* = 0.016), although the interval from the vaccine/infection and the gestational age at amniocentesis were similar for the two groups.

Among women in the vaccine-only group, the median antibody level was higher among those who had received three rather than one or two vaccine doses (274.5, 22.2, and 49.9 AU/mL, respectively, *p* < 0.001). A significant positive correlation was found between the number of vaccine doses and antibody levels (R = 0.488, *p* < 0.001). Furthermore, the rate of positive antibody levels was higher among women who received three compared with one or two doses (72.4%, 18.2%, and 19.2%, respectively, *p* < 0.001).

In a sub-group analysis of women who had been vaccinated in the last 6 months before amniocentesis, positive antibody levels were detected among 77.8% of those who received three doses, 25.7% of those who received two doses, and 20.0% of those who received one dose (*p* < 0.001).

Table 3 describes the antibody status in amniotic fluid samples of the four study groups. All of the women in the infection-only group had negative antibody levels. Only one of them had been infected with COVID-19 in the 6 months before amniocentesis. A higher proportion of women had positive antibody levels in the vaccine + infection than the vaccine-only group (63.6% vs. 35.9%, *p* = 0.029). A higher proportion of women had borderline antibody levels in the vaccine-only than the vaccine + infection group (23.9% vs. 4.5%, *p* = 0.043). In the no vaccine/infection group, one woman had borderline antibody levels and one had positive antibody levels.

In a sub-analysis that included only women who were vaccinated/infected more than 6 months prior to amniocentesis, only 1 out of 20 women in the vaccine-only group had positive levels of antibodies. Similarly, only one out of four women in the vaccine/infection group had positive levels of antibodies. No woman in the infection-only group had positive levels of antibodies. The women who had a vaccine/infection in the 6 months prior to the amniocentesis were more likely to have borderline/positive antibody levels (OR = 18.2, 95% CI 7.3–45.3, *p* < 0.001)

### 3.2. The Correlation between Antibody Level and the Interval from Vaccine/Infection

**Figure 2:**
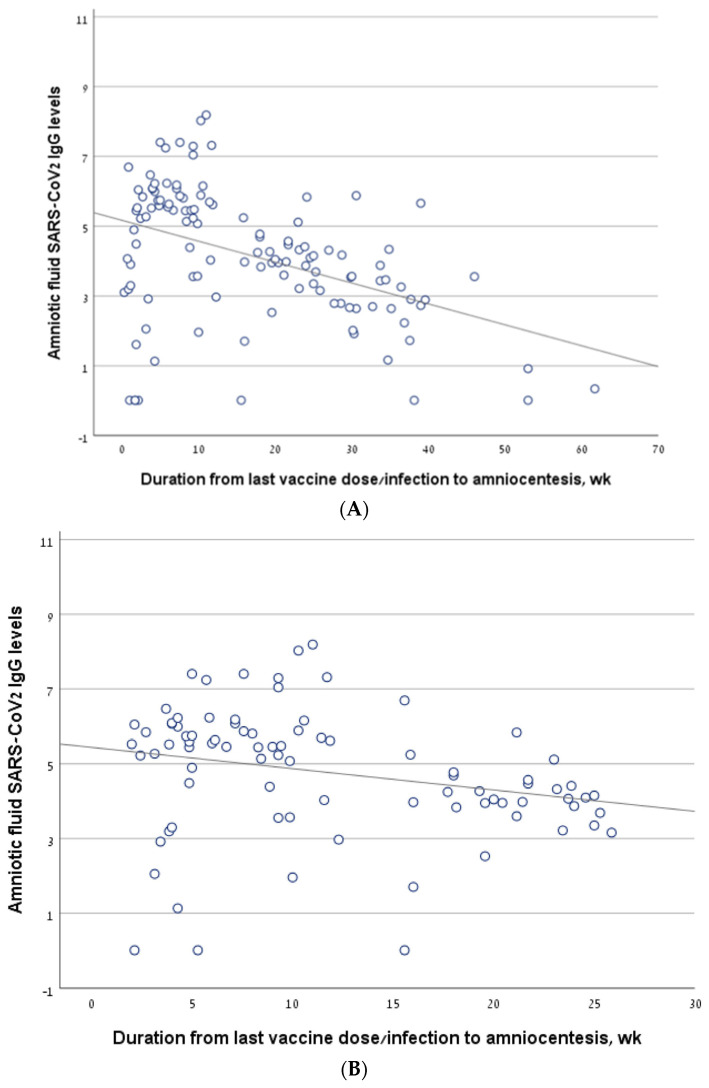

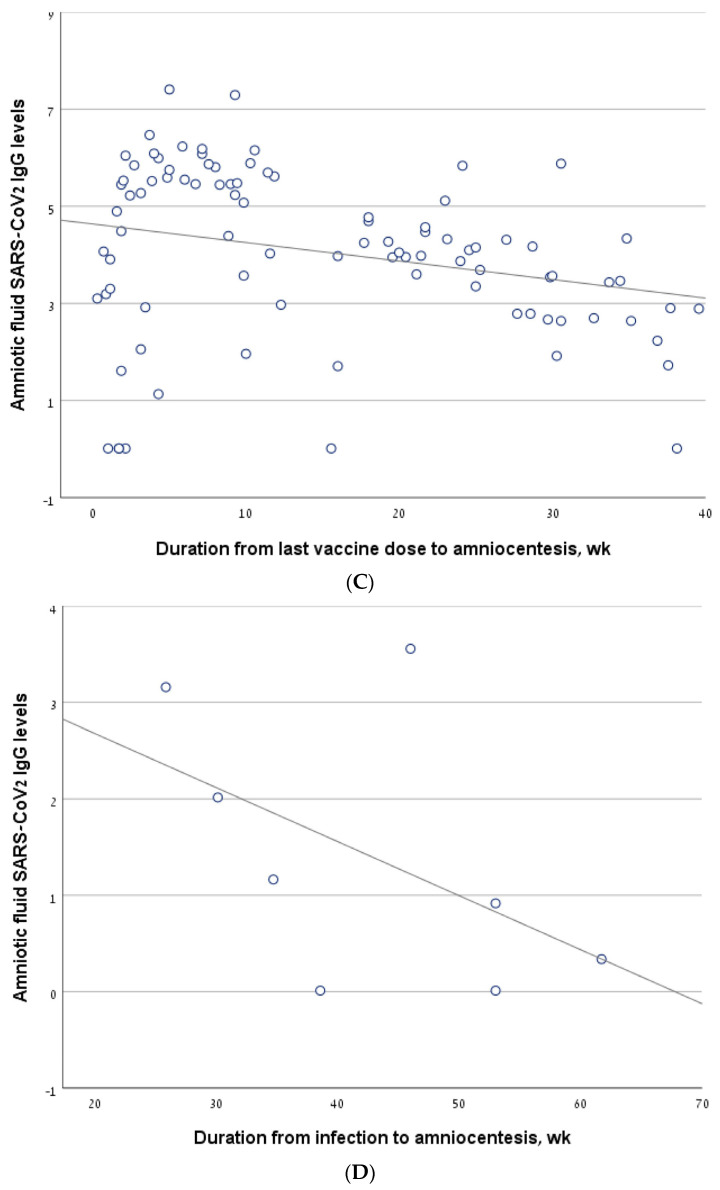

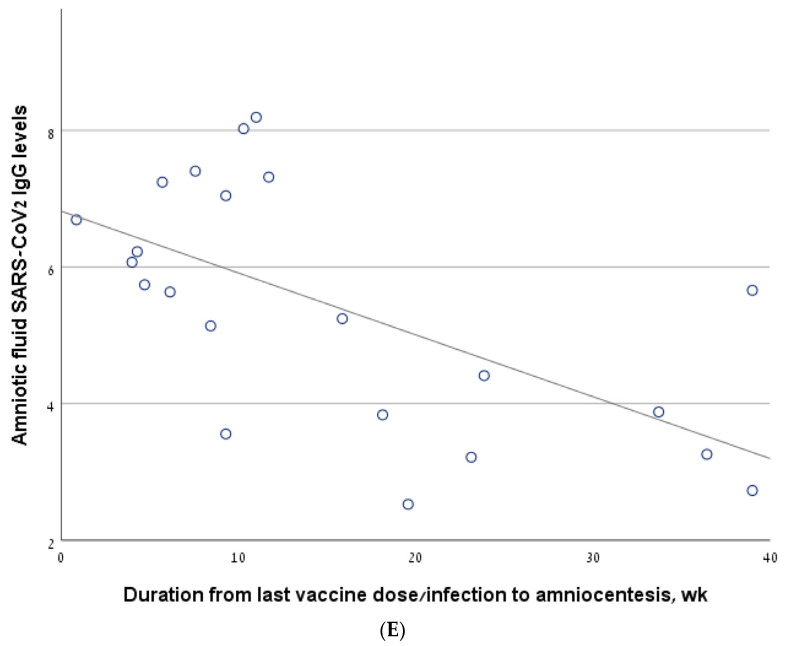
The correlation between antibody levels in amniotic fluid samples of second trimester amniocentesis and the interval from the last vaccine/infection. (**A**) The line describing the correlation, calculated for the entire cohort. (**B**) The line describing the correlation, calculated for a sub-group of women who had been infected and/or vaccinated in the 6 months prior to amniocentesis. (**C**) The line describing the correlation, calculated for the women who had been vaccinated but not infected (vaccine-only group). (**D**) The line describing the correlation, calculated for the women who had been infected but not vaccinated (infection-only group). (**E**) The line describing the correlation, calculated for the women who had been both infected and vaccinated (vaccine + infection group).

Considering all the women who underwent amniocentesis in the second trimester, antibody levels correlated negatively with the interval from the last vaccine/infection to amniocentesis (R = −0.454, *p* < 0.001) (Figure 2A). The findings were similar in a sub-group analysis that included only women who had been infected and/or vaccinated in the 6 months prior to amniocentesis (R = −0.359, *p* = 0.001) (Figure 2B).

The antibody level correlated negatively with the interval from the last vaccine to amniocentesis, both in the vaccine-only group (R = −0.26, *p* = 0.012) (Figure 2C) and in the sub-group of women who were vaccinated in the 6 months before amniocentesis (R = −0.292, *p* = 0.016). For the infection-only group, we did not find a significant correlation between antibody levels and the interval from infection to amniocentesis (R = −0.536, *p* = 0.171) (Figure 2D).

In the vaccine/infection group, we found a significant negative correlation between antibody level and the interval from the last vaccine/infection (−0.559, *p* = 0.007) (Figure 2E). In a sub-analysis of the women who were vaccinated/infected in the 6 months before amniocentesis, the significance was borderline (R = −0.40, *p* value one-sided = 0.05). Thirteen women in the vaccine + infection group had a breakthrough infection after a previous vaccination, while nine women were previously infected and were subsequently vaccinated. The rate of positive antibody levels did not differ between these two sub-groups: 10/13 (76.9%) vs. 4/9 (44.4%), *p* = 0.187.

**Figure 3:**
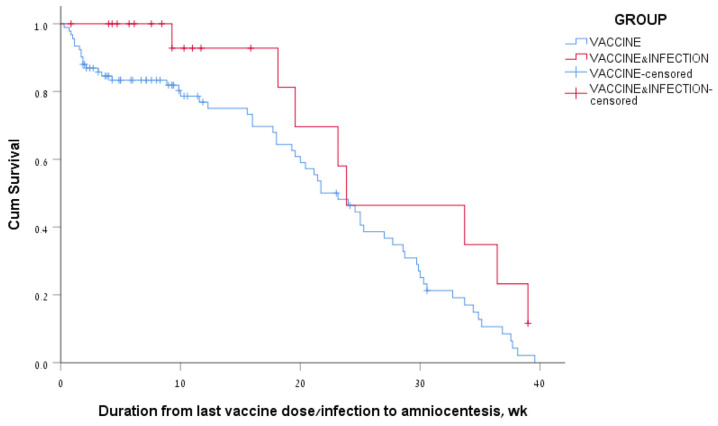
Kaplan–Meier survival curves of amniotic fluid SARS-CoV-2 IgG levels according to the COVID-19 vaccination and infection status of the women.

Among women of the vaccine-only group who had positive antibody levels, 77% had positive antibody levels at 3 months and 34% at 6 months after the last vaccine dose. Among the women of the vaccine + infection group who had positive antibody levels, 93% had positive antibody levels at 3 months and 46% at 6 months after the last vaccine dose/infection. The median survival time for positive SARS-CoV-2 IgG levels in the amniotic fluid was 162 days, a range of 135–189, for the vaccine-only group; and 167 days, a range of 62–272, for the vaccine + infection group (Figure 3). The log rank (Mantel–Cox) *p*-value for comparing survival curves of the two groups was 0.05.

### 3.3. Amniotic Samples of Twin Pregnancies in the Second Trimester

This study included five dichorionic diamniotic twin pregnancies of women who underwent amniocentesis at a median of 18.0 weeks (range 16.7–19.9). One woman had not been infected with COVID-19, nor had she been vaccinated, and no antibodies against SARS-CoV-2 were found in the amniotic fluid sample of either twin. The two other women were vaccinated before the amniocentesis. Both of them received the third vaccine during pregnancy (at 11 and 16 weeks, respectively). The first pair of twins had positive anti-SARS-2 vaccine levels of 435.3 and 433.4 AU/mL, respectively. The other pair of twins did not have antibodies against SARS-CoV-2 in their amniotic samples. Another woman with a twin pregnancy had been infected with COVID-19 at 88.9 weeks before amniocentesis and the amniotic fluid samples of both twins had positive levels, 1869.5 and 619.5 AU/mL. Another woman had been infected with COVID-19 at 39.4 weeks and had received a third vaccine three weeks before amniocentesis. Her twins had borderline antibody levels of 126.6 AU/mL and 270.2 AU/mL.

### 3.4. Amniotic Samples of Amniocentesis in the Third Trimester

The study included 12 women who underwent amniocentesis in the third trimester. The median gestational age at amniocentesis was 31.4 weeks (30.1–34.6). One woman had been infected with COVID-19 at 22.1 weeks prior to amniocentesis and had borderline levels of antibodies of 101.8 AU/mL. Another woman was infected with COVID-19 at 35.9 weeks prior to amniocentesis and had no antibodies in her amniotic fluid sample. Antibodies were not detected in the amniotic fluid sample of one woman who had not been vaccinated or infected. Nine women were vaccinated at 0.7–41.9 weeks before amniocentesis. Two of them had positive and one had borderline antibody levels. No correlation was found between the interval from the last vaccine dose/infection and antibody levels (R = −0.300, *p* = 0.185).

### 3.5. Comment

#### 3.5.1. Principal Findings

We found that SARS-CoV-2 IgG levels were higher among women who had been both vaccinated and infected with the virus than among women who had only been vaccinated. Among women who had received three, compared to one or two, vaccine doses during the 6 months before the second trimester amniocentesis, the median antibody level was higher and the proportion with positive antibody levels was greater. Among women who had only been vaccinated compared to those who had been vaccinated and infected with COVID-19, antibody levels progressively decreased. The duration of time until antibody levels were negative was shorter among women who had only been vaccinated than among those who had been vaccinated and infected with COVID-19.

#### 3.5.2. Results in the Context of What Is Known

Our results of higher antibody levels among vaccinated women with previous COVID-19 infection corroborates Ali et al.’s study of fully vaccinated persons [12]. They reported a steeper slope of decline in antibodies among those without a previous COVID-19 infection than among those with a previous COVID-19 infection. The authors hypothesized that vaccines and infections present the viral protein in slightly different conformations, and that this can yield differences in antigen kinetics and the antibodies produced [13]. Furthermore, the sum of antibodies after a combination of infection and vaccination is higher than after vaccination only. This can also explain our observation of higher antibody levels after three vaccine doses compared to one or two doses [14].

Our results of waning antibody levels after vaccination against SARS-CoV-2 or COVID-19 infection are in line with previous studies that showed limited durability of SARS-CoV-2 antibodies. Siller et al. showed a reduction by 44.0% in anti-spike IgG levels at 5–6 months compared with 0–3 months after infection. In fully vaccinated individuals, the levels decreased by 31.7% per month [15].

Because of waning immunity and decreased vaccine efficacy against circulating variants, and a worse COVID-19 course in pregnant women, a number of international health organizations have recommended a booster dose in pregnancy [9,16]. Atyeo et al. reported significantly increased IgG1 levels against the Omicron variant of COVID-19 following an mRNA booster dose during the third trimester and concordance in maternal and cord IgG1 levels to the ancestral strain and the Omicron spike. These results suggest that a third trimester booster vaccine increases maternal and neonatal immunity against SARS-CoV-2 and its Omicron variant [17]. Notably, in the sub-group of our women who underwent amniocentesis in the third trimester, antibody levels were not found to be correlated to the interval from the last vaccine dose or infection. Nevertheless, the inclusion of only 12 women in this sub-group precludes drawing conclusions regarding amniotic fluid SARS-CoV-2 IgG levels in the third trimester. The American College of Obstetrics and Gynecology recommends that women in early pregnancy and up to 6 weeks postpartum receive a bivalent mRNA COVID-19 vaccine booster dose following the completion of their last COVID-19 primary vaccine dose or monovalent booster [16]. Our results strengthen these recommendations in light of the progressive decrease in immunity against SAR-CoV-2. Nevertheless, our results raise question as to whether two booster doses should be administered in fully vaccinated/previously infected women. The first dose would be in early pregnancy to maintain immunity throughout pregnancy. The second booster dose would be in the third trimester to augment immune protection in mothers and their neonates.

An interesting finding is that positive antibody levels were found in the amniotic fluid of 3.7% of the women in our study who had no history of maternal vaccination or infection. We assume that this result reflects maternal asymptomatic COVID-19 infection [18].

Our conclusions regarding twin pregnancies are limited due to the inclusion of only five pairs of dichorionic–diamniotic twins. The effectiveness of placental transfer has not been fully evaluated in twin pregnancy [16].

#### 3.5.3. Clinical Implications

By including a large number of amniotic fluid samples, we attempted to shed light on maternal immunity and maternal–fetal transfer of antibodies against SARS-CoV-2 in the periconceptional and early pregnancy periods. Understanding the durability of maternal and fetal antibodies and their immune response will guide the need for additional boosters in the future trajectory of the pandemic. Our results raise the question as to whether two boosters are needed during pregnancy, the first at the beginning of pregnancy and the second in the third trimester, to maintain immunity. However, these results should be interpreted with caution, and further research is needed to confirm the most effective vaccination strategies and to elucidate the potential risks involved. Moreover, it is important to recognize that elevated SARS-CoV-2 IgG levels may pose potential risks to newborns, including renal failure, as indicated by recent studies [19]. This highlights the necessity of careful monitoring and balancing the benefits of maternal antibody transfer with the possible adverse outcomes. Notably, therapeutic options, such as statins, have been shown to enhance vaccine responses through their anti-inflammatory and endothelial-protective effects and to reduce adverse outcomes including kidney failure. Such treatments could play a role in mitigating risks and are considered safe during the third trimester of pregnancy [20,21,22]. These benefits likely arise from the capability of statins to dampen excessive inflammatory responses and to support vascular health. In addition to boosting vaccine efficacy, this may protect against complications like kidney injury, thus contributing to better outcomes for both mother and child.

#### 3.5.4. Research Implications

Large prospective studies are needed to examine the optimal vaccination timing for SARS-CoV-2 during pregnancy and the number of booster doses required in pregnant women to supply effective and longstanding immunity, particularly against new variants of SARS-CoV-2. Further research should also explore how medications such as statins, through their pleiotropic effects, including anti-inflammatory, antioxidant, and endothelial-stabilizing actions, might modulate IgG responses and reduce neonatal risks, thus optimizing both maternal and neonatal outcomes.

#### 3.5.5. Strengths and Limitations

The retrospective design is a limitation of this study. For example, some women were not included because they did not have residual specimens of amniotic fluid. Moreover, this study did not include data on pregnancy outcomes, as not all the women delivered at our medical center. Additionally, we were unable to determine whether antibody levels in amniotic fluid are correlated to risks of maternal or neonatal COVID-19 infection. The levels of the SARS-CoV-2 IgG were determined in amniotic fluid samples and not in maternal or cord blood, which might have different immune dynamics. Although most of the women included likely received the Pfizer-BioNTech vaccine, with some probably receiving Moderna [23], we suspect that this variation in vaccine type did not significantly impact the overall antibody responses observed in our study. This is because of the similar immunogenicity reported for the two vaccines. Nevertheless, this was the first study to investigate SARS-CoV-2 IgG levels in a large number of amniotic fluid samples of second trimester amniocentesis. For two previously described fully vaccinated women who underwent amniocentesis at 18 weeks of gestation, anti-SARS-CoV-2 IgG titers in amniotic fluid mirrored the levels detected in the serum and were inversely linked to the time from vaccination [10]. Among 22 women in their second trimester who were either vaccinated against SARS-CoV-2 or had been infected with the virus within the last year, La Fauci et al. found that antibody levels in maternal serum and amniotic fluid correlated significantly [24]. This suggests, as in our study, that antibody transfer may occur early in pregnancy.

## 4. Conclusions

Anti-SARS-CoV-2 antibodies were found in the amniotic fluid of a considerable proportion of pregnant women after vaccination, infection, or both. This could reflect antibody transfer during the peri-conceptional and early pregnancy periods. Our results indicate a trend in progressively decreasing immunity, as reflected in the decreasing amniotic fluid antibody levels in vaccinated, infected, and vaccinated + infected pregnant women. However, further prospective studies on pregnant women are needed to determine the optimal vaccination timing and the number of boosters needed against new variants of SARS-CoV-2.

## Figures and Tables

**Table 1:** Characteristics of women who underwent amniocentesis during the second trimester.

	N = 149
Maternal age (years), median (range)	37 (22–46)
Gravity, median (range)	3 (1–10)
Parity, median (range)	3 (1–7)
ART, n. (%)	16 (10.7)
Medical conditions, n. (%)	17 (11.4)
Medications, n. (%)	16 (10.7)
Body mass index (kg/m^2^), median (range)	28.1 (18.5–44.9)
GA at amniocentesis (weeks), median (range)	19.1 (16.3–26.9)
Time from the last vaccine/infection to amniocentesis (weeks), median (range)	11.0 (0.3–39.6)
Vaccinated/infected in the 6 months before amniocentesis, n. (%)	91 (61.1)
Time from the last vaccine/infection to amniocentesis (only for the women who were vaccinated/infected within the 6 months preceding amniocentesis) (weeks), median (range)	9.3 (0.3–25.9)
One vaccine dose, n. (%)	17 (11.4)
Two vaccine doses, n. (%)	62 (41.6)
Three vaccine dosed, n. (%)	35 (23.5)

ART, artificial reproductive technology; GA, gestational age.

**Table 2:** Characteristics of the women who underwent amniocentesis in the second trimester, according to COVID-19 vaccine and infection status.

	Vaccine Only (N = 92)	Infection Only (N = 8)	Vaccine + Infection (N = 22)	No Vaccine/Infection (N = 27)	*p* Value
Maternal age (years), median (range)	37 (22–46)	38 (32–42)	36 (25–46)	36 (22–46)	1.00
Gravity, median (range)	1 (1–10)	2.5 (1–8)	3 (2–10)	2 (1–9)	0.202
Parity, median (range)	1 (1–7)	2 (1–7)	3 (1–5)	2 (1–6)	0.394
ART, n. (%)	10 (11.0)	1 (12.5)	2 (10.5)	3 (11.1)	1.00
Medical conditions, n. (%)	11 (12.0)	0 (0)	4 (18.2)	2 (7.4)	0.603
Medications, n. (%)	10 (10.9)	0 (0)	2 (9.1)	4 (14.8)	0.808
Body mass index (kg/m^2^), median (range)	28.1 (18.5–44.9)	32.9 (25.1–35.5)	30.1 (21.0–38.3)	25.6 (19.3–37.9)	0.290
GA at amniocentesis (weeks), median (range)	19.3 (16.3–26.9)	18.0 (16.6–21.3)	19.8 (17.3–21.9)	18.1 (17.0–22.3)	0.522
Time from the last vaccine/infection to amniocentesis (weeks), median (range)	11.0 (0.3–39.6)	42.3 (25.9–61.7)	10.7 (0.9–39.0)	-	<0.001 ^ǂ^ 0.699
Vaccinated/infected in the 6 months before amniocentesis, n. (%)	72 (78.3) ^#^	1 (12.5)	18 (81.8) ^#^		<0.001
Time from the last vaccine/infection to amniocentesis (only for the women who were vaccinated/infected within the 6 months preceding amniocentesis) (weeks), median (range)	8.9 (0.3–25.3)	22.1 *	9.8 (4.0–23.9)	-	0.09 ^ǂ^
SARS-CoV2 IgG levels, median (range)	64.1 (0–1643.7)	2.9 (0–35.0)	283.0 (12.5–3603.3)	17.9 (0–267.8)	<0.001
Number of vaccine doses received before amniocentesis					0.206
1 vaccine dose, n. (%)	11 (12.0)	-	6 (27.3)	-	
2 vaccine doses, n. (%)	52 (56.5)	-	10 (45.5)	-	
3 vaccine doses, n. (%)	29 (31.5)	-	6 (27.3)	-	

^ǂ^ Comparison between the vaccine-only and vaccine + infection groups; ^#^ *p* value = 1.00. * Only one woman had COVID-19 in the 6 months before amniocentesis.

**Table 3:** Antibody status in amniotic fluid samples, according to the COVID-19 vaccination and infection status of the women.

	Vaccine Only (N = 92)	Infection Only (N = 8)	Vaccine + Infection (N = 22)	No Vaccine/Infection (N = 27)	*p* Value
Negative antibodies, n. (%)	37 (40.2)	8 (100)	7 (31.8)	25 (92.6)	<0.001 ^ǂ^ 0.627
Borderline antibodies, n. (%)	22 (23.9)	0 (0)	1 (4.5)	1 (3.7)	0.014 ^ǂ^ 0.043
Positive antibodies, n. (%)	33 (35.9)	0 (0)	14 (63.6)	1 (3.7)	<0.001 ^ǂ^ 0.029

^ǂ^ Comparison between the vaccine-only and vaccine + infection groups.

## Data Availability

The datasets analyzed during the current study are available from the corresponding author on reasonable request.

## References

[1] McClymont E., Albert A.Y., Alton G.D., Boucoiran I., Castillo E., Fell D.B., Kuret V., Poliquin V., Reeve T., Scott H. (2022). Association of SARS-CoV-2 Infection during Pregnancy with Maternal and Perinatal Outcomes. JAMA.

[2] Martinez-Portilla R.J., Sotiriadis A., Chatzakis C., Torres-Torres J., Sosa S.E.Y., Sandoval-Mandujano K., Castro-Bernabe D.A., Medina-Jimenez V., Monarrez-Martin J.C., Figueras F. (2021). Pregnant women with SARS-CoV-2 infection are at higher risk of death and pneumonia: Propensity score matched analysis of a nationwide prospective cohort (COV19Mx). Ultrasound Obstet. Gynecol..

[3] Kleinwechter H.J., Weber K.S., Mingers N., Ramsauer B., Schaefer-Graf U.M., Groten T., Kuschel B., Backes C., Banz-Jansen C., Berghaeuser M.A. (2022). Gestational diabetes mellitus and COVID-19: Results from the COVID-19-Related Obstetric and Neonatal Outcome Study (CRONOS). Am. J. Obstet. Gynecol..

[4] Smith E.R., Oakley E., Grandner G.W., Rukundo G., Farooq F., Ferguson K., Baumann S., Waldorf K.M.A., Afshar Y., Ahlberg M. (2023). Clinical risk factors of adverse outcomes among women with COVID-19 in the pregnancy and postpartum period: A sequential, prospective meta-analysis. Am. J. Obstet. Gynecol..

[5] Stock S.J., Carruthers J., Calvert C., Denny C., Donaghy J., Goulding A., Hopcroft L.E.M., Hopkins L., McLaughlin T., Pan J. (2022). SARS-CoV-2 infection and COVID-19 vaccination rates in pregnant women in Scotland. Nat. Med..

[6] Prasad S., Kalafat E., Blakeway H., Townsend R., O’brien P., Morris E., Draycott T., Thangaratinam S., Le Doare K., Ladhani S. (2022). Systematic review and meta-analysis of the effectiveness and perinatal outcomes of COVID-19 vaccination in pregnancy. Nat. Commun..

[7] Rottenstreich A., Zarbiv G., Oiknine-Djian E., Vorontsov O., Zigron R., Kleinstern G., Wolf D.G., Porat S. (2022). The Effect of Gestational Age at BNT162b2 mRNA Vaccination on Maternal and Neonatal Severe Acute Respiratory Syndrome Coronavirus 2 (SARS-CoV-2) Antibody Levels. Clin. Infect. Dis..

[8] Yang Y.J., Murphy E.A., Singh S., Sukhu A.C.B., Wolfe I.B., Adurty S.B., Eng D.B., Yee J.B., Mohammed I.B., Zhao Z. (2022). Association of Gestational Age at Coronavirus Disease 2019 (COVID-19) Vaccination, History of Severe Acute Respiratory Syndrome Coronavirus 2 (SARS-CoV-2) Infection, and a Vaccine Booster Dose with Maternal and Umbilical Cord Antibody Levels at Delivery. Obstet. Gynecol..

[9] COVID-19 Vaccination Considerations for Obstetric–Gynecologic Care. https://www.acog.org/clinical/clinical-guidance/practice-advisory/articles/2020/12/covid-19-vaccination-considerations-for-obstetric-gynecologic-care.

[10] Colavita F., Oliva A., Bettini A., Antinori A., Girardi E., Castilletti C., Vaia F., Liuzzi G. (2022). Evidence of Maternal Antibodies Elicited by COVID-19 Vaccination in Amniotic Fluid: Report of Two Cases in Italy. Viruses.

[11] Narasimhan M., Mahimainathan L., Araj E., Clark A.E., Markantonis J., Green A., Xu J., SoRelle J.A., Alexis C., Fankhauser K. (2021). Clinical evaluation of the abbott alinity SARS-CoV-2 spike-specific quantitative igg and igm assays among infected, recovered, and vaccinated groups. J. Clin. Microbiol..

[12] Ali H., Alahmad B., Al-Shammari A.A., Alterki A., Hammad M., Cherian P., Alkhairi I., Sindhu S., Thanaraj T.A., Mohammad A. (2021). Previous COVID-19 Infection and Antibody Levels After Vaccination. Front. Public Health.

[13] Greaney A.J., Loes A.N., Gentles L.E., Crawford K.H., Starr T.N., Malone K.D., Chu H.Y., Bloom J.D. (2021). Antibodies elicited by mRNA-1273 vaccination bind more broadly to the receptor binding domain than do those from SARS-CoV-2 infection. Sci. Transl. Med..

[14] Desmecht S., Tashkeev A., El Moussaoui M., Marechal N., Perée H., Tokunaga Y., Fombellida-Lopez C., Polese B., Legrand C., Wéry M. (2022). Kinetics and Persistence of the Cellular and Humoral Immune Responses to BNT162b2 mRNA Vaccine in SARS-CoV-2-Naive and -Experienced Subjects: Impact of Booster Dose and Breakthrough Infections. Front. Immunol..

[15] Siller A., Seekircher L., Wachter G.A., Astl M., Tschiderer L., Pfeifer B., Gaber M., Schennach H., Willeit P. (2022). Seroprevalence, Waning and Correlates of Anti-SARS-CoV-2 IgG Antibodies in Tyrol, Austria: Large-Scale Study of 35,193 Blood Donors Conducted between June 2020 and September 2021. Viruses.

[16] COVID-19 Vaccines and Pregnancy: Conversation Guide. https://www.acog.org/covid-19/covid-19-vaccines-and-pregnancy-conversation-guide-for-clinicians.

[17] Atyeo C., Shook L.L., Nziza N., Deriso E.A., Muir C., Baez A.M., Lima R.S., Demidkin S., Brigida S., De Guzman R.M. (2023). COVID-19 booster dose induces robust antibody response in pregnant, lactating, and nonpregnant women. Am. J. Obstet. Gynecol..

[18] Yefet E., Massalha M., Alter A., Harnik A.G., Mahamed S.H., Novick L., Wattad M., Sakas J., Baram S., Weiss A. (2023). Should pregnant women be screened for SARS-CoV-2 infection? A prospective multicenter cohort study. Int. J. Gynaecol. Obstet..

[19] Pawar R., Gavade V., Patil N., Mali V., Girwalkar A., Tarkasband V., Loya S., Chavan A., Nanivadekar N., Shinde R. (2021). Neonatal Multisystem Inflammatory Syndrome (MIS-N) Associated with Prenatal Maternal SARS-CoV-2: A Case Series. Children.

[20] Black S., Nicolay U., Del Giudice G., Rappuoli R. (2016). Influence of Statins on Influenza Vaccine Response in Elderly Individuals. J. Infect. Dis..

[21] Piani F., Di Salvo E., Landolfo M., Saracino I.M., Agnoletti D., Borghi C., Fiorini G. (2023). Statin therapy may protect against acute kidney injury in patients hospitalized for interstitial SARS-CoV2 pneumonia. Nutr. Metab. Cardiovasc. Dis..

[22] Torres-Peña J.D., Pérez-Belmonte L.M., Fuentes-Jiménez F., Carmona M.D.L., Perez-Martinez P., Lopez-Miranda J., Sanchez F.J.C., Nunez J.A.V., Beamonte E.d.C., Gamboa J.O.M. (2021). Prior Treatment with Statins is Associated with Improved Outcomes of Patients with COVID-19: Data from the SEMI-COVID-19 Registry. Drugs.

[23] COVID-19 Vaccination Information—Ministry of Health. https://corona.health.gov.il/vaccine-for-covid/.

[24] La Fauci L., Cavaliere R., Romeo P., Alibrandi A., Ferlazzo G., D’Anna R., Corrado F. (2023). Anti-Severe Acute Respiratory Syndrome Coronavirus 2 IgG Is Present in the Amniotic Fluid of both Infected and Vaccinated Women at Second Trimester of Pregnancy: A Cohort Study. Fetal. Diagn. Ther..

